# Polymerase chain reaction detection of genes responsible for multiple antibiotic resistance *Staphylococcus aureus* isolated from food of animal origin in Egypt

**DOI:** 10.14202/vetworld.2017.1205-1211

**Published:** 2017-10-09

**Authors:** Fawzy R. El Seedy, A. A. Samy, Hala S. H. Salam, Eman A. Khairy, Aya A. Koraney

**Affiliations:** 1 Department of Bacteriology, Mycology and Immunology, Faculty of Veterinary Medicine, Beni-Suef University, Beni-Suef, Egypt; 2 Department of Microbiology and Immunology, National Research Center, Cairo, Egypt

**Keywords:** food of animal origin, multiple antibiotic resistance, polymerase chain reaction, resistance genes, *Staphylococcus aureus*

## Abstract

**Aim:**

The aim of our study was polymerase chain reaction (PCR) detection of the genes responsible for the multiple antibiotic resistance *S. aureus* isolated from food of animal origin in Egypt.

**Materials and Methods:**

A total of 125 samples were randomly collected from milk, meat, and their products from Giza and Beni-Suef Governorates markets. The *S. aureus* isolates were subjected to antimicrobial sensitivity tests using four antibacterial disks (Oxoid), and then the polymerase chain reaction (PCR) was performed for detection of antibiotic resistance genes.

**Results:**

Out of 125 samples, 19 *S. aureus* isolates were detected. All detected isolates were multiple drug resistance (MDR). The penicillin-, erythromycin-, kanamycin-, and tetracycline-resistant isolates were examined by PCR for resistance genes *blaZ*, (*msrA, ermB*, and *ermC)*, *aac(6’)aph (2”)*, and *tetK*. The isolates harbored these resistance genes with percentage of 100% (100%, 0%, and 100%), 62.5%, and 100%, respectively.

**Conclusion:**

Contaminated foods of animal origin may represent a source of MDR *S. aureus* that can be a major threat to public health.

## Introduction

The development of multiple antibiotic-resistant bacteria due to misuse of antibiotics in animals and poultry production is well authenticated for pathogenic bacteria [1,2]. Contaminated food of animal origins with antibiotic-resistant bacteria can be a great threat to public health, and the antibiotic resistance determinants can be transferred from antibiotic-resistant bacteria to other bacteria affecting human [3,4]. Identical elements of antibiotic-resistant genes found in bacteria that affect both animals and humans have shown the role of raw foods in the dissemination of these resistance genes through the food chains [5,6] or through occupational contact with livestock [7].

The multiple antibiotic-resistant bacteria were commonly isolated from food of animal origin such as raw milk and unpasteurized dairy products [2,8] and meat products [9], the resistance genes can be transferred from antibiotic-resistant bacteria to the intestinal flora of humans through food products, and the commensally flora can be a reservoir of resistant genes for pathogenic bacteria [10]. The high prevalence of multidrug-resistant *Staphylococcus aureus* was discovered from food of animal origin in Europe, Canada, and United States in multiple studies [11,12], which represents a huge problem in public health [13,14].

The aim of our study was polymerase chain reaction (PCR) detection of the genes responsible for the multiple antibiotic resistance *S. aureus* isolated from food of animal origin in Egypt.

## Materials and Methods

### Ethical approval

No animals were involved in the study at any stage.

### Samples

A total of 125 samples were collected from meat, milk, and their products from Giza and Beni-Suef Governorates markets (Table-1), and all samples were aseptically collected and examined for detection of *S. aureus*.

**Table-1:** Samples collected and their numbers from sale markets.

Product	Milk	Yoghurt	Kareish	Minced meat	Burger	Luncheon	Total
Numbers	28	18	19	20	20	20	125

### Identification and characterization of *S. aureus*

One loopful from prepared incubated samples was plated onto nutrient agar (Difco) and mannitol salt agar (Difco), incubated for 18-24 h at 37°C and examined for bacterial growth. The suspected colonies were identified morphologically and biochemically [15].

### Antimicrobial sensitivity test for identified strains

The antimicrobial sensitivity tests were done by disk diffusion technique [16] using 4 antibacterial disks (kanamycin, penicillin, erythromycin, and tetracycline) - Oxoid - the degree of sensitivity was interpreted according to Koneman *et al.*, and CLSI [17,18].

### PCR detection of the resistance genes

PCR was performed for detection of resistance genes in biotechnology center in the animal health institute according to Sambrook and Russel [19]. The primers were synthesized by Metabion Company, Germany, as mentioned in Table-2.

**Table-2:** Oligonucleotide primers sequences of resistance genes.

Antibiotic resistance	Target gene	Primer sequence (5’-3’)	Length of amplified product (bp)	References
Penicillin	*blaZ*	ACTTCAACACCTGCTGCTTTC	173	[20]
		TGACCACTTTTATCAGCAACC		
Tetracycline	*tet*(K)	GTAGCGACAATAGGTAATAGT	360	
		GTAGTGACAATAAACCTCCTA		
Aminoglycoside	*aac(6’)aph (2”)*	GAAGTACGCAGAAGAGA	491	
		ACATGGCAAGCTCTAGGA		
Erythromycin	*msr*(A)	GCAAATGGTGTAGGTAAGACAACT	400	[21]
		ATCATGTGATGTAAACAAAAT		
	*erm(C)*	ATCTTTGAAATCGGCTCAGG	295	
		CAAACCCGTATTCCACGATT		
	*erm*(B)	CATTTAACGACGAAACTGGC	425	
		GGAACATCTGTGGTATGGCG		

## Results

### Results of recovery rate of *S. aureus* isolates

Out of 125 collected samples, 19 *S. aureus* isolates were detected (Table-3) [20,21].

**Table-3:** Prevalence of the isolated *S. aureus*.

Source of the samples	Total number of samples examined	Recovered *S. aureus* examined/total number of original samples
		n (%)
Milk	28	8 (28.5)
Yogurt	19	1 (5.2)
Kareish cheese	18	0 (0)
Total milk and milk products	65	9 (13.8)
Minced meat	20	5 (25)
Burger	20	2 (10)
Luncheon	20	3 (15)
Total meat and meat products	60	10 (16.6)
Total collected samples	125	19 (15.2)

*S. aureus*=*Staphylococcus aureus*

### Results of antibacterial sensitivity

Results of antibiotic sensitivity test on 19 isolates of *S. aureus* recovered from raw milk, meat, and their products, 14 isolates exhibited resistance against penicillin (73.6%), 11 isolates were resistant against tetracycline (57.8%), while 5 isolates exhibited resistance to erythromycin (26.3%), and 8 isolates were resistant to kanamycin (42.1%) (Table-4).

**Table-4:** Results of antibiotic sensitivity tests.

Antibacterial agent	Milk and milk products (total n=9)			Meat and meat products (total n=10)		
	
	Sensitive	Intermediate	Resistant	Sensitive	Intermediate	Resistant
					
	n (%)	n (%)	n (%)	n (%)	n (%)	n (%)
Penicillin groups						
Penicillin	2 (22.2)	0 (-)	7 (77.7)	3 (30)	0 (-)	7 (70)
Tetracycline group						
Tetracycline	2 (22.2)	0 (-)	7 (77.7)	3 (30)	3 (30)	4 (40)
Aminoglycoside group						
Kanamycin	2 (22.2)	2 (22.2)	5 (55.5)	4 (40)	3 (30)	3 (30)
Macrolide group						
Erythromycin	6 (66.6)	1 (11)	2 (22.2)	5 (50)	2 (20)	3 (30)

### Results of PCR for amplification of *blaz* gene at 173 bp fragment

**Figure-1:**
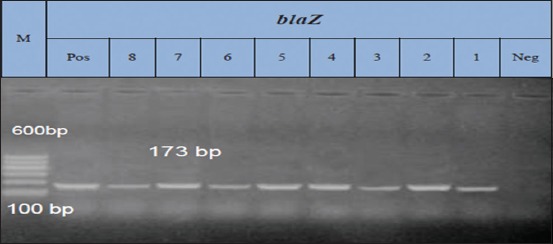
Lane: 1-8 positive amplification of *blaz* gene at 173 bp. Neg=Negative control, pos=Positive control, M=Marker.

Fourteen isolates exhibited resistance against penicillin, out of them; eight isolates were randomly selected for genotypic detection of *bla*z gene. Amplification of *bla*z gene at amplicon size of 173 bp was detected in all the tested isolates (8) with a percentage of 100% (Figure-1).

### Results of PCR for amplification of tet K gene at 360 bp fragment performed with its specific primer

Eleven isolates exhibited resistance against tetracycline, out of them; eight isolates were selected randomly for genotypic detection of *tetK* gene.

**Figure-2:**
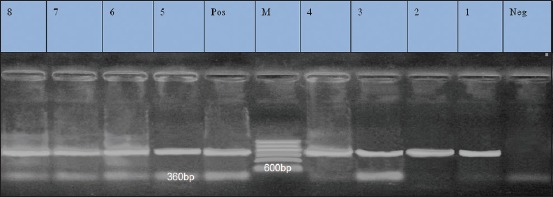
Lane 1-8: Positive amplification of tet K gene at 360 bp. Neg=Negative control, pos=Positive control, M=Marker.

Amplification of *tet*K gene at amplicon size of 360 bp was detected in all the tested isolates with a percentage of 100% (Figure-2).

### Results of PCR for amplification of *ermB* (425 bp), *msrA* (400 bp), and *ermC* (295 bp) for erythromycin resistance isolates

**Figure-3:**
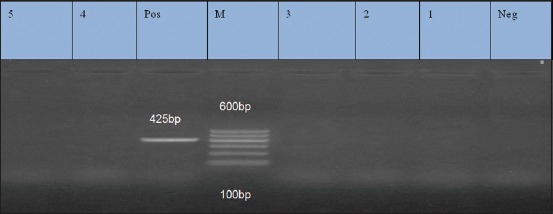
Lane 1, 2, 3, 4, 5: Negative amplification of *ermB* gene at 425 bp. Neg=Negative control, Pos=Positive control, M=Marker.

**Figure-4:**
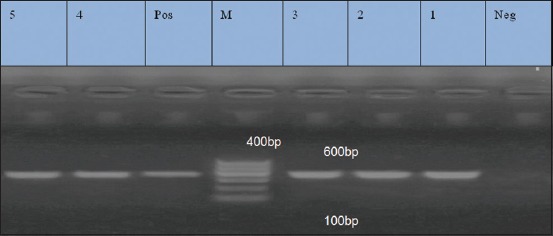
Lane 1-5: Positive amplification of *msrA* gene at 400 bp. Neg=Negative control, pos=Positive control, M=Marker.

**Figure-5:**
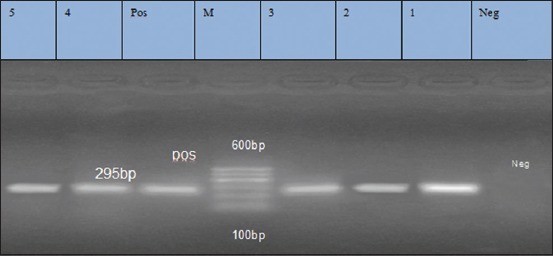
Lane 1-5: Positive amplification of *ermC* gene at 295 bp. Neg=Negative control, pos=Positive control, M=Marker.

Five isolates were resistant to erythromycin. They were examined by PCR for detection of *erm*B, *msr*A, and *erm*C genes. They exhibited prevalence of 0%, 100%, and 100% for *erm*B, *msr*A, and *erm*C genes, respectively (Figures-3-5).

### Results of PCR for amplification of 491 bp fragment for *aac(6’) aph (2”)* gene (aminoglycoside)

**Figure-6:**
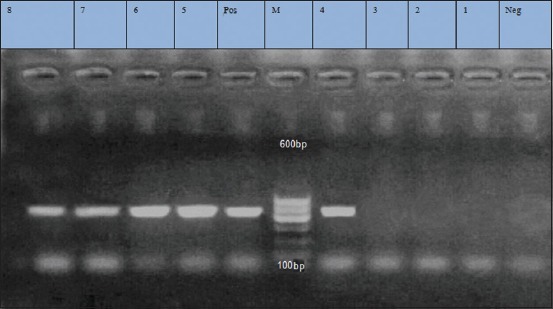
Lane 4, 5, 6, 7, 8 positive amplification of aac(6’) aph (2”) at 491 bp. Lane 1, 2, 3: Negative amplification of aac (6’) aph (2”). Neg=Negative control, pos=Positive control, M=Marker.

Antibiotic susceptibility against aminoglycoside (kanamycin) using disk diffusion method revealed that eight isolates were resistant to kanamycin. They were examined by PCR for detection of *aac*(6’) *aph*(2”) gene, and the result revealed that the *aac*(6’) *aph*(2”) was detected in five (62.5%) out of eight isolates (Figure-6).

## Discussion

The livestock products could be a source of exposure to multidrug-resistant *S. aureus* strains as a result of hazardous misuse of antibiotics in animal treatment and unhygienic livestock practices [2]. Food of animal origin is an ideal culture medium for growth of many organisms [22]. They are considered as a shelter of different types of microorganisms through processing, handling, preparation, and storage as well as distribution [23]. They are considered as major sources of foodborne diseases and have been linked to serious outbreaks of food poisoning worldwide.

The result showed in Table-3 reported that *S. aureus* isolated from raw milk and milk products (Kareish cheese and yoghurt), and from meat and meat products (burger and luncheon) were 13.8% and 16.6%, respectively. A higher recovery rate was obtained by El-Jakee *et al*. [24], they isolated *S. aureus* from cow milk (22.7%) and buffalo milk (16%), and on the other hand, El-Jakee *et al*. [25] revealed that raw milk contaminated with *S. aureus* with an incidence of 56%. While Jamali *et al*. [26] reported *S. aureus* with a percentage of 15.7% and 12.4% from dairy products and raw milk, respectively, Imani *et al*. [27] found 4% of milk and 32% of dairy products contaminated with *S. aureus*, similar results were obtained by Song *et al*. [28], who isolated *S. aureus* from raw milk with a percentage of 20.2%. Higher results of *S. aureus* contamination were reported in raw milk by Gwida and El-Gohary [29]. They recorded 56.66% of *S. aureus* present in market milk. The prevalence of *S. aureus* in Kareish cheese in our results is lower than the result reported by Hosny *et al*. [30], who isolated *S. aureus* with a percentage of 17% from milk shops and street vend but reported the same result from brand cheese. The presence of *S. aureus* in milk was variable in different regions, and these variations may be due to season, number of animals on the farm, farm size, hygiene status, variation in sampling, farm management practices, geographical location, and differences in detection methods and variation in types of samples evaluated. El-Sayed *et al*. [31] revealed that in Egypt the difference in white soft cheese due to acidity as Domiati or Kareish acid coagulation, enzyme coagulation, keeping temperatures, different salt concentrations, and ripening in brine solutions are factors affecting the microbiological quality of these varieties.

The incidence of *S. aureus* in meat products in our study agrees with the findings of the study by Fox *et al*. [32], who tested 124 raw meat samples for methicillin-resistant *S. aureus* (MRSA) including pig (n=63), poultry (n=50), and turkey (n=11) collected from England between March and July 2015. MRSA was isolated from 9 (73%) samples (4 poultry, 3 pig, and 2 turkeys) from different butchers and supermarkets. While Pesavento *et al*. [33] isolated *S. aureus* from raw meat 23.86%. Another study by Ge *et al*. [34] detected *S. aureus* from retail meats of turkey, pork, beef, and chickens. While Ali *et al*. [35] isolated *S. aureus* from meat samples with a percentage of 7%. Hassanin [36] isolated *S. aureus* from burger and luncheons agreed with our results with a percentage of 25% and 47.5%, respectively, but differ in minced meat (65%). *S. aureus* was isolated from meat products with a percentage of 30% by Abdaslam *et al*. [37]. While Li *et al*. [38] were recorded 27.9% of *S. aureus* isolates from food sample. Another study recovered *S. aureus* in 27 from 165 retail meat samples with a percentage of 16.4% [39]. On the other hand, Song *et al*. [28] isolated *S. aureus* 21.3% from frozen food and 28.1% from raw meat samples.

Transmission of antibiotic-resistant *S. aureus* strains can be done by contaminated foods with resistant bacteria [40]. Some researchers reported a primary relationship between the prevalence of antibiotic-resistant bacteria and the misusing of antibiotics for therapeutic purposes in animals [41].

The result of our *S. aureus* sensitivity test (Table-4) revealed that 14 out of 19 identified *S. aureus* isolates were resistant to penicillin (73.6%), while 11 isolates exhibited resistance against tetracycline (57.8%), 5 isolates were resistant to erythromycin (26.3%), and 8 isolates were resistant to kanamycin (42.1%). All the isolates were multidrug resistance (MDR) because they were resistant for more than one antibiotic class. The same results were found by Ammar *et al*. [42], and they observed MDR *S. aureus* (MDRSA) among 85% of isolates recovered from examined milk and meat product samples. Approximately 10.4% from *S. aureus* detected in retail meats in 1-year survey (2010-2011) collected from eight U.S. states were MDRSA Ge *et al*. [34]. While Jamali *et al*. [26] stated that 36.3%, 46.6%, and 12.8% of isolates were resistant to one, two, and more than two antimicrobial agents, respectively, and found that *S. aureus* resistant to tetracycline with a percentage of 56.1%, chloramphenicol (3.7%), and gentamicin (2.1%) but low incidence in case of erythromycin, kanamycin, streptomycin, penicillin G, and oxacillin. Our study agreed with many reports indicating a high percentage of multidrug-resistant *S. aureus* isolates from food of animal origin [32,43-45]. The same results obtained by Argudín *et al*. [46], and they found that *S. aureus* resistant to oxacillin (95%) and trimethoprim-sulfamethoxazole (4%) but differed in erythromycin (70%), tetracycline (100%), kanamycin (29%), and gentamicin (14%) and also reported that 4%, 30%, 21%, and 33% of *S. aureus* isolates were resistant to more than four, three, and five classes of antibiotics, respectively. Tan *et al*. [47] disagreed with our results, and they stated that 94.59% of the strains were resistant only to one of the antibiotics or did not resistant to all of the tested antibiotics, while only 5.41% of *S. aureus* strains were multidrug resistant, while Teramoto *et al*. [48] found that *S. aureus* isolates from conventional retail meat were resistant to both erythromycin 50.0% and tetracycline 58.3%.

PCR-based molecular methods are preferred for determination of antibiotic-resistant genes. Recently, many studies have demonstrated the extremely high capacity of molecular methods such as PCR and pulsed-field gel electrophoresis; these methods were increasingly used for their specific, rapid, reliable, and accurate detection of bacteria and genes of interest [49]. Nowadays, the detection of antibiotic-resistant genes was accomplished by PCR methods directed to the linA, *tetK*, *msrA*, msrB, ermA, *ermC*, *aacA-D*, and *tetM* gens [50]. In this work, PCR primers that can be used to survey clinically relevant antibiotic resistance genes frequently encountered in *S. aureus*.

The results revealed that all tested isolates of *S. aureus* which were resistant to penicillin carried *blaZ* genes, the erythromycin-resistant isolates carried *ermB*, *msrA*, and *ermC* with a percentage of 0%, 100%, and 100%, respectively, and aminoglycoside gene *aac(6’) aph (2”)* (kanamycin resistance gene) present in a percentage of 62.5% in the resistant isolates while the tetracycline-resistant isolates carried *tetK* gene with a percentage of 100%. Our findings agreed with McCallum *et al*. and Argudín *et al*. [46,51], they reported that *tetK* gene was present in a percentage of 91% of tetracycline resistant, 70% of erythromycin-resistant isolates carried resistance genes (encoded by *ermC*, ermA, and *ermB*, alone or in combination), and *aac(6’)aph* (2”) gene found in kanamycin-resistant gene, while penicillin-resistant isolates carried *blaZ* gene 94%. While Jamali *et al*. [26] found that *blaZ* (97.4%) and *tetK* (41.8%) present in penicillin- and tetracycline-resistant isolates, respectively, and *msrA* and *ermC* genes (erythromycin resistance gene) present in high prevalence as our results but with different prevalence of *ermB* gene. The high prevalence of the *blaZ* and tet M resistance genes in this study is in agreement with the results reported by Gao *et al*. [52]. The same findings were obtained by Li *et al*. [38], on *msrA*, *ermC*, *tetK*, and *blaZ* genes with different results for *ermB*. Duran *et al*. [20] reported erythromycin resistance genes (*ermA*, *ermB*, *ermC*, and *msrA*), one of them at least was present in erythromycin-resistant isolates, *tetM* or *tetK* or both resistance genes isolates were found in tetracycline-resistant isolates, *aac(6’)aph(2”)* presents in gentamicin susceptible *S. aureus* isolates, and major of staphylococci tested possessed the *blaZ* gene (89.9%). Argudín *et al*. [46] reported that all strains resistant to ampicillin–penicillin carried the *blaZ* gene, and they detected the genes responsible for erythromycin resistance together with inducible resistance to clindamycin were *ermA* and *ermC* while resistance to erythromycin only was associated with the presence of either *msrB* or *msrB msrA*. A high prevalence of *ermB* gene than *ermC* in food of animal origin was detected by Martineau *et al*. [53].

## Conclusion

Foods of animal origin may represent a source of MDR *S. aureus* that can be a major threat to public health infection for humans.

## Authors’ Contributions

FRE and AAS have planned the research work; also they participate laboratory work with HSHS, EAK, and AAK. All authors.
